# Spectral efficient and high data rate ring topology based UWOC system with carrier-free optical add-drop nodes

**DOI:** 10.1371/journal.pone.0345092

**Published:** 2026-03-24

**Authors:** Ammar Armghan, Slim Chaoui, Meshari Alsharari, Khaled Aliqab, Salman Ghafoor, Muhammad Ijaz, Jawad Mirza

**Affiliations:** 1 Department of Electrical Engineering, College of Engineering, Jouf University, Sakaka, Saudi Arabia; 2 Department of Computer Engineering and Networks, College of Computer and Information Sciences, Jouf University, Sakaka, Saudi Arabia; 3 School of Electrical Engineering and Computer Science, National University of Sciences and Technology, Islamabad, Pakistan; 4 Department of Engineering, Manchester Metropolitan University, Manchester, United Kingdom; 5 Department of Electrical Engineering, HITEC University Taxila, Pakistan; 6 SEECS Photonics Research Group, Islamabad, Pakistan.; Beijing Institute of Technology, CHINA

## Abstract

In this paper, we report a ring topology based spectral efficient and high data rate underwater wireless optical communication (UWOC) system transmitting full-duplex data to five different optical add-drop nodes (OADNs) distributed in the ring. The UWOC system is designed so that all five nodes can transmit uplink (UL) channels without requiring optical source. The combined optical signal composed of five different wavelengths each modulated by the downlink (DL) data at the rate of 20 Gb/s using quadrature phase shift keying (QPSK) is transmitted towards the nodes. The DL baseband data is recovered at respective nodes using homodyne detection and the same optical carrier is re-used to transmit the UL data at the rate of 20 Gb/s encoded using on-off keying (OOK) modulation format towards central unit (CU). A specially designed pulse carver based on dual drive Mach-Zehnder modulator (DD-MZM) is employed to implement the transmission of full-duplex data in the ring. Each node in the ring operates at data rate of 20 Gb/s and is located at a fixed distance of 25 m from the next node in the ring. Underwater bubbling induced turbulence is modelled using Gamma-Gamma channel model. The performance of the DL and UL channels is evaluated using Bit-error rate (BER) results obtained for different generation rates of underwater bubbles. The simulation results clearly indicate that target forward-error correction (FEC) BER limit of 3.8 × 10^−3^ is achieved for both DL and UL channels under different bubble generation rates. The failure model introduced in proposed study confirms that the system sustains 80% node connectivity under single OADN failure and 100% node connectivity under primary link failure through link redundancy without changing the path inflation. These findings reflect that the proposed ring topology based UWOC system is flexible and resilient to the adverse channel effects, making it a promising solution for high-speed, long-range future Internet of underwater things (IoUT) applications.

## 1 Introduction

Recent years have witnessed a surge of interest in marine exploration, driving the advancement of new high-speed wireless communication systems to meet the demands of real-time applications. In this context, underwater wireless communication (UWC) is vital for ocean exploration and development which mainly exploits wireless carriers such as radio frequency (RF) waves, acoustic waves, and optical waves to transmit the data in aquatic environments [1]. Among these technologies, underwater wireless optical communication (UWOC) has attracted significant attention due to its ability to deliver higher data rates with lower latency compared to acoustic communication while overcoming the high underwater attenuation associated with RF links [2]. Additional advantages include enhanced security, improved anti-interference capability, and lower operational costs [1,2]. As a result, UWOC is increasingly becoming a primary solution for a variety of commercial and scientific applications including undersea rescue, disaster response, ocean monitoring, and exploration [3]. Apart from numerous benefits, the underwater environment is complex and dynamic which makes the UWOC links easily interfered by various factors such as oceanic turbulence, underwater attenuation, and pointing errors [3]. Among these adverse factors, bubbles-induced oceanic turbulence is a crucial factor which affects the reliability of the UWOC links [3,4].

Ring topology based telecommunication networks are typically valued for their resilience and efficiency, offering redundant and full-duplex data transmission that enhance fault tolerance and simplify the network management. This architecture has a well-established history in terrestrial communications having been extensively implemented and researched in fiber-optic communication systems [5] and recently adapted for free-space optical (FSO) communication [6] to implement robust and high-capacity networks. However, its application remained largely unexplored in the underwater domain until recently. The emergence of UWOC access systems now marks a significant advancement in marine connectivity, offering high-speed and low-latency data transmission where traditional methods fall short. By adapting this proven ring-based architecture, UWOC systems leverage its inherent robustness and reliability, interconnecting multiple nodes in a closed loop to ensure redundant paths for data transmission and significantly enhance resilience against frequent link failures caused by the challenging underwater channels. Such ring topologies are particularly suited for real-time monitoring systems, underwater sensor networks, and collaborative autonomous vehicle operations, ultimately paving the way for more scalable and dependable underwater networks by enabling efficient and fault-tolerant communication.

Bubbles in the ocean can originate from various sources such as the movement of marine organisms, sea divers, unmanned underwater vehicles (UUVs), decomposition of inorganic matter, and leaks from submarine gas fields [2]. The presence of underwater bubbles in the UWOC channel cause variation in the underwater refractive index and increases the probability of bubble-beam interactions, thus resulting into beam misalignment, beam wandering, spot dancing, and intensity fluctuations at the photodetector [7]. The presence of bubbles in the direction of beam propagation greatly increases the outage probability by obstructing the line-of-sight (LOS) connection between the transmitter and receiver telescopes [8]. Therefore, it is crucial to characterize the effect of underwater bubbles on the channel to design a reliable UWOC system.

In recent years, various researchers have conducted their research work on UWOC systems. For instance, Cai et al. demonstrated a Gb/s level real-time full-duplex UWOC transceiver based on laser diodes [9], Fan et al. developed a high-speed long-distance UWOC system based on laser diodes and OOK modulation [10], Li et al. demonstrated a real-time, multi-user UWOC system for DL transmission based upon arrayed light emitting diodes (LEDs) and field programmable gate arrays (FPGAs) exploiting non-orthogonal multiple access (NOMA) [11], Wang et al. proposed a real-time duplex UWOC system based on blue/green laser diodes and a high-sensitive multi-pixel photon counters (MPPC) utilizing 4-quadrature amplitude modulation (4QAM) and orthogonal frequency division multiplexing (OFDM) [12], Salman et al. proposed a machine learning assisted underwater wireless sensor network based on multiple diversity gain-enabled relays for full-duplex transmission of data between the nodes and gateway [13], Li et al. proposed autonomous underwater vehicles (AUVs) based UWOC system for UL transmission using NOMA to enhance the spectral efficiency [14], Hang et al. proposed a secure UL hybrid RF-UWOC system that consists upon a number of underwater sensors, a floating relay node, a legitimate satellite receiver, and an eavesdropping satellite [15], Zhang et al. proposed a reconfigurable intelligent surface (RIS) assisted UWOC system for DL transmission based on intensity modulation and direct detection (IM-DD) [16], Nawal et al. demonstrated red vertical-cavity surface-emitting lasers (VCSELs) based UWOC link for data transmission employing direct-current-biased orthogonal frequency-division multiplexing (DC-OFDM) modulation [17], and Naik et al. proposed a simultaneous transmit and reflect (STAR)-RIS enabled UWOC system allowing multiple users to transmit their data simultaneously in all directions [18]. We have added Table 1 that highlights the main findings of the past studies discussed above to further elaborate the literature review.

**Table 1:** Literature review and main findings.

Study	Rate	Modulation	Range	Directionality	Wavelength
[9]	1.2 Gb/s	OOK	30 m	Full-duplex	525 nm
[10]	10 Mb/s	OOK	105 m	Full-duplex	450 nm
[11]	40 Mb/s	OOK	–	Downlink	450 nm
[12]	25 Mb/s	4QAM	50 m	Duplex	450 nm
[13]	0.5 Mb/s	OOK	10 m	Full-duplex	470 nm
[14]	–	OOK	–	Uplink	–
[16]	–	OOK	50 m	Downlink	530 nm
[17]	2 Gb/s	NRZ-OOK,DC-OFDM	1 m	Downlink	645 nm
[18]	–	OOK, BPSK, QAM	4 m	Omnidirectional	470 nm

**Fig 1:**
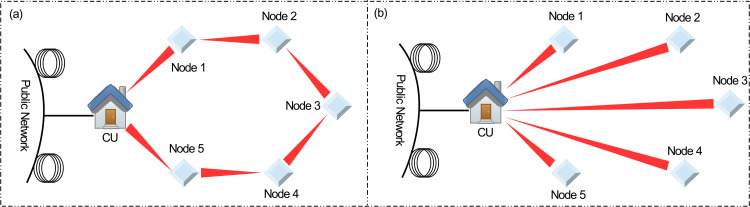
Illustration of UWOC systems exploiting. **(a)** Ring topology **(b)** Tree topology.

In this work, we propose to the best of our knowledge for the first time a UWOC ring topology designed to transmit data across five symmetrically spaced OADNs, each separated by a distance of 25 m. This approach enables full-duplex transmission of QPSK and OOK modulated DL and UL channels, respectively at the rate of 20 Gb/s. Two key contributions of this architecture are (I) Design of a pulse carver based upon a sinusoidal RF source and DD-MZM for continuous wave (CW) to pulsed signal conversion that is employed at the CU to transmit the DL and UL channels simultaneously (II) Implementation of carrier-free nodes achieved by leveraging two distinct modulation formats to reuse the DL optical carriers originating from the CU for UL transmission. The performance of DL and UL channels is analyzed using BER results and eye diagrams for different generation rates of underwater bubbles considering Gamma-Gamma channel model. The advantages of the proposed architecture are illustrated in Fig 1, which compares the ring topology with an alternative tree topology.

As shown in Fig 1a, the ring architecture requires only a single pair of telescopes at the CU to handle the transmission of five DL and UL channels. In contrast, the conventional tree topology in Fig 1b necessitates five pairs of telescopes. This reduction in required hardware significantly lowers the capital expenditure (CAPEX), making the ring topology based UWOC link more cost-effective solution. Similarly, the proposed architecture offers various other advantages beyond the cost efficiency. As shown in Fig 1b, in a conventional tree topology, nodes 2 and 3 are located far from the CU making them highly susceptible to oceanic turbulence and attenuation. These channel impairments often degrade the received signal quality to such an extent that FEC limit cannot be achieved. In contrast, the ring topology enables robust connectivity to all nodes by incorporating optical amplifiers at each OADNs. Each OADN not only receives and transmits DL and UL channels respectively but also functions as an amplify and forward optical relay (AFOR), effectively extending the total range of the link while maintaining signal integrity. Another key advantage of the ring topology is enhanced reliability. In a tree structure, each node depends entirely on a single direct link to the CU. If any transmitter or receiver fails, that node experiences a complete outage. The ring architecture, however, provides redundant bidirectional pathways allowing data to travel in clockwise or counterclockwise directions. If one segment fails, traffic can be diverted to the opposite direction, ensuring continuous operation. This inherent fault tolerance makes the ring topology especially suitable for applications where high reliability is critical. The research work carried out in this paper has been implemented employing “OptiSystem 22” simulation software developed by Optiwave Inc [19]. The remainder of this paper is organized as follows. Section-2 details the signal transmission scheme and turbulence model. Section-3 presents the design of the pulse carver, while Section-4 describes the proposed system architecture. The ring’s failure model is introduced in Section-6 and results are discussed in Section-6. Finally, Section-7 provides the conclusion.

## 2 UWOC signal transmission and turbulence model

### 2.1 UWOC signal transmission model

Accurate channel modelling is essential in UWOC studies, as it governs the behavior of light propagation through aquatic environment. Primary phenomena affecting signal integrity include absorption, scattering, and turbulence. The absorption coefficient (a(λ)) characterizes energy loss during light propagation while the scattering coefficient (b(λ)) describes the photon redirection due to interaction with water constituents or suspended matter. The total attenuation coefficient is represented as [20].


c(λ)=a(λ)+b(λ) (1)

Where, *λ* is the operating wavelength. The total signal attenuation follows the Beer-Lambert law [20].


$$I=I_{0}\exp\left[-c(\lambda )\cdot L\right]$$ (2)

Here, *I*_0_ denotes the initial irradiance, c(λ) is the total attenuation coefficient, *L* is the length of UWOC channel in water, and *I* is the resulting irradiance after propagation.

### 2.2 UWOC Turbulence Model

In addition to the absorption and scattering losses, the intensity of the received optical signal at photodetector undergoes random fluctuations caused by the turbulence, called intensity scintillation [21]. The scintillation index (SI) serves as a key metric for quantifying the intensity fluctuations of the received optical signal propagating through UWOC channel. It is mathematically defined as follows [20].


$$\sigma_{I}^{2}=\frac{𝔼[I^{2}]-(𝔼[I]{)}^{2}}{(𝔼[I]{)}^{2}}$$ (3)

Where, $\sigma_{I}^{2}$ is the SI, 𝔼[I] is the expected value of the light intensity *I*, and $𝔼[I^{2}]$ is the expected value of the squared intensity. In UWOC systems, the SI serves as a critical indicator of turbulence-induced signal degradation. A lower SI value signifies minimal intensity fluctuations indicating stable channel conditions with reduced turbulence impact on the communication link. On the other hand, a higher SI reflects strong turbulence effects resulting in significant signal fading and degraded transmission quality. This parameter is essential for evaluating channel quality, designing effective fading mitigation strategies, and optimizing overall system performance in UWOC systems.

The random fluctuation in received signal’s intensity is commonly modeled using the Gamma-Gamma distribution [21]. This model is well-suited for estimating turbulence-induced fading in UWOC systems, as it accounts for both small-scale and large-scale eddies [21]. The presence of underwater bubbles significantly alters the oceanic turbulence. The bubble-induced oceanic turbulence is quantified through the refractive index structure parameter $C_{n}^{2}$, which is characterized by void fraction parameter (Γ) [22].


$$C_{n}^{2}=8\pi \beta_{oc}\cdot 0.033\cdot (n_{w}-n_{a}{)}^{2}\cdot \frac{\langle \Gamma^{2}\rangle }{L_{0}^{2/3}}$$ (4)

where, $\beta_{oc}\approx 0.4$ is the Obukhov-Corrsin constant, $n_{w}-n_{a}=0.341$ is the refractive index contrast, $L_{0}\approx 0.1$ m is the outer scale of turbulence, and $\langle \Gamma^{2}\rangle$ is the mean-square void fraction fluctuation. The void fraction variance ($\langle \Gamma^{2}\rangle$) can be related to the bubble generation rate (bubbles·m^−3^·s^−1^) through conservation of bubble number. Assuming monodisperse bubbles of radius *a* = 100 *μ*m and a characteristic dissolution time $\tau_{d}\approx 30$ s for oceanic conditions [23].


$$\langle \Gamma^{2}\rangle \approx {\left(B\cdot \frac{4}{3}\pi a^{3}\cdot \tau_{d}\right)}^{2}$$ (5)

Where, *B* is the bubble generation rate. Substituting Eq. 5 into Eq. 4, direct relationship between bubble generation rate and oceanic turbulence can be obtained.


$$C_{n}^{2}\approx 8\pi \beta_{oc}\cdot 0.033\cdot (n_{w}-n_{a}{)}^{2}\cdot \frac{{\left(B\cdot \frac{4}{3}\pi a^{3}\cdot \tau_{d}\right)}^{2}}{L_{0}^{2/3}}$$ (6)

The $C_{n}^{2}$ values in Table 2 are calculated using Eq. 6 for different bubble generation rates corresponding to all oceanic regimes.

**Table 2:** Bubble generation rates, corresponding optical turbulence parameters, and derived air flow rates for different oceanic regimes. Values calculated from Eqs. 4-10 assuming monodisperse 100 *μ*m bubbles with $\tau_{d}=30$ s.

Bubble Induced Turbulence	Bubble Generation Rate	Air Flow Rate
Weak bubbling ($10^{-16}$ m^−2/3^)	$2.0\times 10^{7}$ *Bubbles*/*L*_*w*_ *min*	0.084 $L_{a}/L_{w}min$
Moderate bubbling ($10^{-14}$ m^−2/3^)	$2.0\times 10^{8}$ *Bubbles*/*L*_*w*_ *min*	0.84 $L_{a}/L_{w}min$
Strong bubbling ($10^{-12}$ m^−2/3^)	$2.0\times 10^{9}$ *Bubbles*/*L*_*w*_ *min*	8.4 $L_{a}/L_{w}min$

The corresponding Gamma-Gamma parameters (*α* and *β*) are then obtained by substituting $C_{n}^{2}$ into the standard expressions for a plane wave [24].


$$\sigma_{1}^{2}=1.23C_{n}^{2}k^{7/6}L^{11/6}$$ (7)

$$\alpha ={\left[\exp\left(\frac{0.49\sigma_{1}^{2}}{(1+1.11\sigma_{1}^{12/5}{)}^{7/6}}\right)-1\right]}^{-1}$$ (8)

$$\beta ={\left[\exp\left(\frac{0.51\sigma_{1}^{2}}{(1+0.69\sigma_{1}^{12/5}{)}^{5/6}}\right)-1\right]}^{-1}$$ (9)

Where, k=2π/λ is the optical wavenumber and *L* is the UWOC link range. The airflow rate ${\text{F}}_{air}$ in Table 2 is derived from bubble generation rate assuming the same monodisperse bubble population.


$$F_{\text{air}}=Q\cdot \frac{4}{3}\pi a^{3}$$ (10)

Eq. 10 represents the air volume injected per unit volume of water per unit time. Finally, the modified probability density function (pdf) of the received intensity as a function of bubble generation rate is given by.


$$p(I|B)=\frac{2(\alpha (B)\beta (B){)}^{(\alpha (B)+\beta (B))/2}}{\Gamma (\alpha (B))\Gamma (\beta (B)))}\cdot I^{\frac{\alpha (B)+\beta (B)}{2}-1}\cdot K_{\alpha (B)-\beta (B)}\left(2\sqrt{\alpha (B)\beta (B)I}\right)$$ (11)

Where, Γ(·) is the Gamma function and $K_{\nu }(\cdot )$ is the modified Bessel function of the second kind.

Different underwater bubble generation rates are considered in OptiSystem to analyze the performance which are characterized by air flow rate as shown in Table 2.

The mapping from bubble generation to optical turbulence follows established fluid dynamics principles where bubbles act as passive scalars in turbulent flow. The void fraction fluctuations are mixed by turbulent eddies following Kolmogorov-Obukhov-Corrsin scaling, resulting to refractive index variations proportional to $(n_{w}-n_{a}{)}^{2}\cdot \frac{\langle \Gamma^{2}\rangle }{L_{0}^{2/3}}$ as given by Eq. 6. The selected parameters correspond to documented oceanic conditions. Bubble radii of 100 *μ*m represent the dominant persistent size range in breaking waves [23]. The dissolution time $\tau_{d}=30$ s matches the lifetime of such bubbles in seawater. The bubble generation rates span realistic regimes as, 2 × 10^7^ $L_{w}^{-1}$ min^−1^ (weak) corresponds to background wind-driven mixing, 2 × 10^8^ $L_{w}^{-1}$ min^−1^ (moderate) to visible whitecapping, and 2 × 10^9^ $L_{w}^{-1}$ min^−1^ (strong) to active wave breaking during storms. The resulting $C_{n}^{2}$ values from $10^{-16}$ to $10^{-12}$ m^−2/3^ cover the measured range of oceanic optical turbulence from calm to highly bubbly waters [24], ensuring the analysis captures realistic underwater channel conditions. Therefore, the parameter mapping establishes a physically interlinked sequence: increased bubble generation leads to greater void fraction variance, which in turn produces stronger refractive index fluctuations, ultimately resulting in higher levels of optical turbulence.

## 3 Working principle of the proposed pulse carver

**Fig 2:**
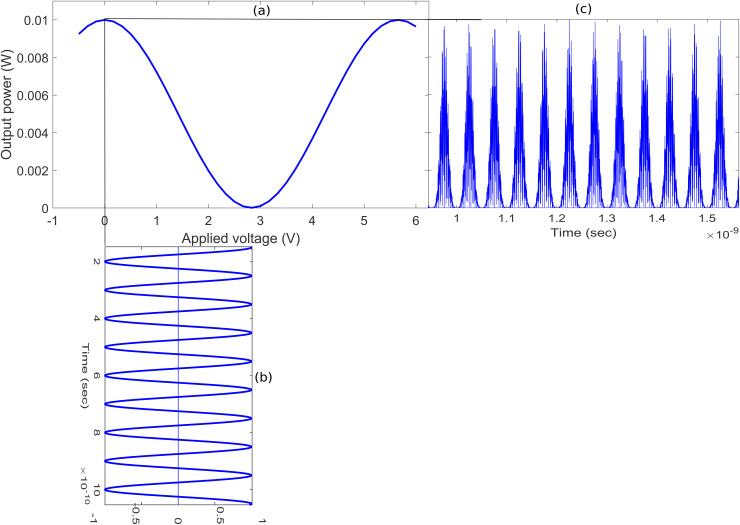
Working principle of the pulse carver. **(a)** DD-MZM transfer function **(b)** Normalized plot of applied sinusoidal RF signal **(c)** Normalized plot of DL data modulated pulsed optical signal.

Fig 2 explains the operating principle of pulse carver based on DD-MZM and sinusoidal RF source employed in the proposed architecture for CW to pulse signal conversion to implement the transmission of full-duplex data in the ring. The output optical field *E*_*o*_(*t*) for an input optical field *E*_*i*_(*t*) is expressed as [25].


$$E_{o}(t)=\frac{E_{i}(t)}{2}\left[\exp\left(j\frac{\pi V_{1}(t)}{V_{\pi }}\right)+\exp\left(j\frac{\pi V_{2}(t)}{V_{\pi }}\right)\right]$$ (12)

Where, $\alpha_{i}$, *P*_*T*_, and $P_{Y}/P_{T}$ denote the insertion loss, total optical power, and power splitting (or combining) ratio of the two modulator arms, respectively. Additionally, *v*_1_, *v*_2_, and $v_{\pi }$ represent the effective drive voltages applied to the modulator arms and the switching voltage of the modulator, respectively. The transfer characteristic of the DD-MZM depicting the output power as a function of applied input voltage is presented in Fig. 2a. To transform the CW signal into pulsed form, the DD-MZM is biased at its maximum transmission point and driven by a sinusoidal electrical RF signal as shown in Fig. 2b that switches it between its minimum and maximum transmission states. In this work, the sinusoidal drive signal operates at a frequency of 10 GHz, which corresponds to half of the target data rate (i.e., 20 Gb/s). This configuration generates optical pulses with a 33% duty cycle and 20 GHz repetition rate as shown in Fig. 2c.

## 4 Proposed architecture

**Fig 3:**
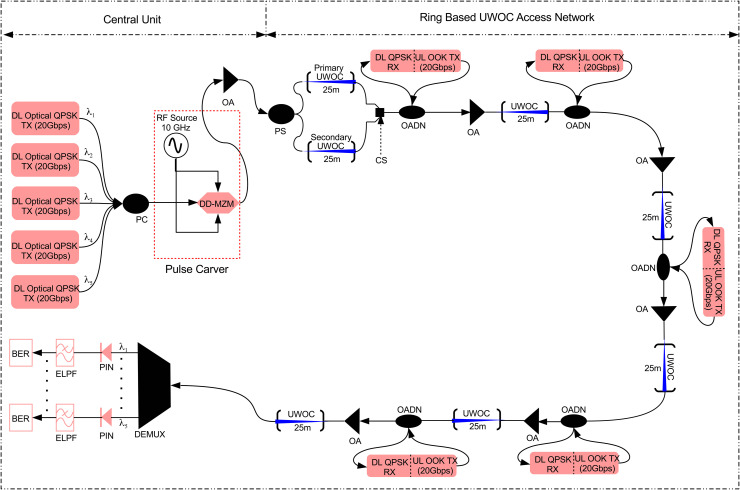
Block diagram of the proposed architecture. **TX/RX**: Transmitter/receiver, **DD-MZM**: Dual-drive Mach-Zehnder modulator, **OA**: Optical amplifier, **UWOC**: Underwater wireless optical communication channel, **OADN**: Optical add-drop node, **DEMUX**: Demultiplexer, **PS**: Power splitter, **PIN**: Positive interinsic negative photodetector, **ELPF**: Electrical lowpass filter, **BER**: Bit-error rate estimator.

**Fig 4:**
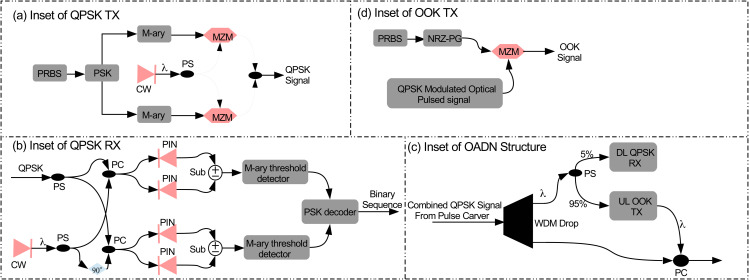
Detailed structure of. **(a)** QPSK transmitter **(b)** QPSK receiver **(d)** OADN **(d)** OOK transmitter, **PRBS**: Pseudo random bit sequence generator, **NRZ-PG**: Non-return to zero pulse generator, **PSK**: Phase shift keying, **MZM**: Mach-Zehnder modulator, **CW**: Continuous wave laser, **PC**: Power combiner, **PS**: Power splitter, **Sub**: Subtractor, **PIN**: Positive interinsic negative photodetector.

Fig 3 illustrates the physical layer design of the proposed UWOC ring architecture. It may be observed that at the CU, five optical QPSK transmitters each having powers of 25 dBm and wavelengths of $\lambda_{1}=532\;nm$, $\lambda_{2}=532.8\;nm$, $\lambda_{3}=533.6\;nm$, $\lambda_{4}=534.4\;nm$, and $\lambda_{5}=535.2\;nm$ are available to transmit QPSK encoded DL data of five OADNs, each at the rate of 25 Gb/s. It is pertinent to mention here that each OADN is separated by a distance of 25 m. The detailed structure of each optical QPSK transmitter has been shown in Fig 4a.

**Fig 5:**
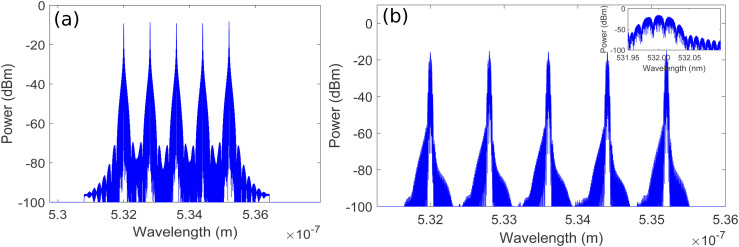
Spectral plots of combined. **(a)** Optical QPSK signal at the input of the pulse carver **(b)** Pulsed signal at the output of pulse carver after CW to pulse conversion.

The optical QPSK signals are combined using an optical power combiner (PC). The combined optical signal at an aggregated data rate of 100 Gb/s is then given as input to a pulse carver to convert the CW signals into pulsed optical signals with 33% duty cycle and 20 GHz repetition rate as already discussed in Section-3. The spectral plots of combined optical QPSK signal at the input of pulse carver and pulsed signal of five OADNs at the output of pulse carver after CW to pulse conversion have been shown in Fig 5. It is evident from inset of Fig 5b that multiple coherent sidebands are generated around the center wavelength of 532 nm, which are located at multiples of sinusoidal RF source’s frequency.

**Fig 6:**
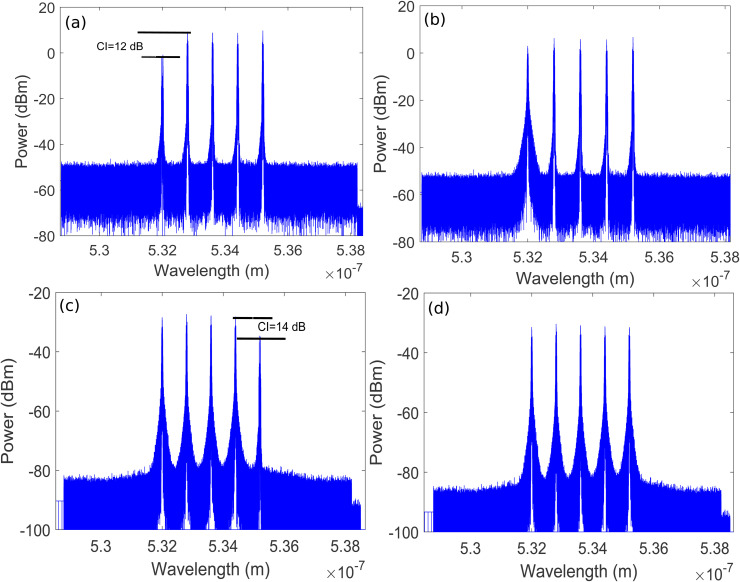
Spectral plots of. **(a)** QPSK modulated DL channel at dropped at OADN-1 **(b)** OOK modulated UL channel at added at OADN-1 **(c)** QPSK modulated DL channel at dropped at OADN-5(d) OOK modulated UL channel at added at OADN-5.

The resulted DL data modulated optical pulsed signals of five OADNs are amplified and forwarded to a transmitter telescope. The gain of the optical amplifier (OA) is adjusted such that the combined data modulated pulsed optical signal of all five OADNs at the input of the transmitter telescope has an average power of 47.4 dBm. Using the transmitter telescope, the combined pulsed signal is propagated over the first UWOC channel having range of 25 m. The UWOC channel is modeled by the Gamma-Gamma distribution as discussed in Section-2, which imparts distortions due to bubbling induced turbulence and underwater attenuation. A receiver telescope at OADN-1 then receives the combined pulsed signal. Fig 4c illustrates the detailed internal structure of an OADN. It may be observed that the incoming signal is given as input to a wavelength division multiplexing drop (WDMD) componenet where a particular channel from a WDM signal is dropped and allowed to pass others. Therefore, QPSK modulated DL channel at $\lambda_{1}=532\;nm$ is dropped at OADN-1. Fig 6a shows the spectral plot of DL channel at $\lambda_{1}=532\;nm$ dropped at OADN-1 with channel isolation (CI) of around 12 dB.

The QPSK modulated DL channel at $\lambda_{1}=532\;nm$ is further split into two parts using a 95:5 power splitter (PS) as shown in Fig 4c. As illustrated in Fig 3, 5% of the PS output is directed to a QPSK receiver for DL data recovery, while the remaining 95% is remodulated with UL data using OOK format. The detailed structure of UL OOK transmitter is shown in Fig 4d. At the receiver, QPSK modulated DL channel at $\lambda_{1}=532\;nm$ is photodetected based on the principle of homodyne detection. The detailed structure of optical QPSK receiver is shown in Fig 4c. The electrical signals (inphase and quadrature components) at the output of the detectors are low-pass filtered. After PSK decoding, the retrieved binary data of DL channel are passed to the BER estimator for BER calculation as shown in Fig 3. The BER is determined by analyzing the statistical parameters of the received signal’s eye-diagram. QPSK modulation is employed for DL data transmission as it modulates only the optical phase while maintaining intensity. Consequently, the same QPSK modulated optical carrier is intensity-modulated with 20 Gb/s OOK-encoded UL data, as illustrated in Fig 3. The OOK modulated UL channel at $\lambda_{1}=532\;nm$ and the remaining four QPSK modulated DL channels at $\lambda_{2}=532.8\;nm$, $\lambda_{3}=533.6\;nm$, $\lambda_{4}=534.4\;nm$, and $\lambda_{5}=535.2\;nm$ are recombined using a PC as shown in Fig 4c and amplified using an OA. Fig 6b shows the spectral plot of the combined optical signal of OOK modulated UL channel at $\lambda_{1}=532\;nm$ and the remaining four QPSK modulated DL channels at $\lambda_{2}=532.8\;nm$, $\lambda_{3}=533.6\;nm$, $\lambda_{4}=534.4\;nm$, and $\lambda_{5}=535.2\;nm$. The gain of OA is adjusted such that the combined optical signal at the input of the transmitter telescope has a power of 38.4 dBm. This combined signal is transmitted towards OADN-2 having a range of 25 m. The combined optical signal composed of one OOK modulated UL channel and four QPSK modulated DL channels is received at OADN-2 where QPSK modulated DL channel at $\lambda_{2}=532.8\;nm$ is demodulated using the same technique as discussed above for OADN-1. Similarly, the DL channel at $\lambda_{2}=532.8\;nm$ is intensity modulated by the UL data at the rate of 20 Gb/s encoded in OOK format. It is crucial to mention here that OADN-2 also has the same components as OADN-2, as shown in Fig 4. Two OOK modulated UL channels at $\lambda_{1}=532\;nm$ and $\lambda_{1}=532.8\;nm$ alongwith remaining three QPSK modulated DL channels at $\lambda_{3}=533.6\;nm$, $\lambda_{4}=534.4\;nm$, and $\lambda_{5}=535.2\;nm$ are again recombined using a PC and amplified using an OA. The gain of OA is adjusted such that the combined optical signal at the input of the transmitter telescope has a power of 29.5 dBm. This combined signal is transmitted towards OADN-3 having a range of 25 m. The combined optical signal composed of two OOK modulated UL channels and three QPSK modulated DL channels is received at OADN-3 where the QPSK modulated DL channel at $\lambda_{2}=533.6\;nm$ is demodulated. Similarly, the DL channel at $\lambda_{2}=533.6\;nm$ is intensity modulated by the UL data at the rate of 20 Gb/s encoded in OOK format. Three OOK modulated UL channels at $\lambda_{1}=532\;nm$, $\lambda_{1}=532.8\;nm$, and $\lambda_{2}=533.6\;nm$ alongwith remaining two QPSK modulated DL channels at $\lambda_{4}=534.4\;nm$ and $\lambda_{5}=535.2\;nm$ are again recombined using a PC and amplified using an OA. The gain of OA is adjusted such that the combined optical signal at the input of the transmitter telescope has a power of 20.4 dBm. This combined signal is transmitted towards OADN-4 having a range of 25 m. The combined optical signal composed of three OOK modulated UL channels and two QPSK modulated DL channels is received at OADN-4 where the QPSK modulated DL channel at $\lambda_{2}=534.4\;nm$ is demodulated. Similarly, the DL channel at $\lambda_{2}=534.4\;nm$ is intensity modulated by the UL data at the rate of 20 Gb/s encoded in OOK format. Four OOK modulated UL channels at $\lambda_{1}=532\;nm$, $\lambda_{1}=532.8\;nm$, $\lambda_{2}=533.6\;nm$, and $\lambda_{4}=534.4\;nm$ alongwith remaining one QPSK modulated DL channels at $\lambda_{5}=535.2\;nm$ are again recombined using a PC and amplified using an OA. The gain of OA is adjusted such that the combined optical signal at the input of the transmitter telescope has a power of 11.4 dBm. This combined signal is transmitted towards the OADN-5 having a range of 25 m. The combined optical signal composed of four OOK modulated UL channels and one QPSK modulated DL channels is received at OADN-5 where the QPSK modulated DL channel at $\lambda_{2}=535.2\;nm$ is demodulated. Similarly, the DL channel at $\lambda_{2}=535.2\;nm$ is intensity modulated by the UL data at the rate of 20 Gb/s encoded in OOK format. The combined optical signal at the output of OADN-5 carrying OOK modulated UL channels from five OADNs at wavelengths of $\lambda_{1}=532\;nm$, $\lambda_{2}=532.8\;nm$, $\lambda_{3}=533.6\;nm$, $\lambda_{4}=534.4\;nm$, and $\lambda_{5}=535.2\;nm$ is amplified having optical power of 32.3 dBm and transmitted towards the CU over last UWOC channel of 25 m range as illustrated in Fig 3. In this way, a total distance of 150 m has been covered by DL and UL channels over the UWOC ring. At CU, the received combined optical signal is demultiplexed and photodetected. The electrical signals at the output of the photodetectors are lowpass filtered to remove high frequency harmonics and passed on to BER estimators for BER analysis. The main parameters employed in the numerical simulations are listed in Table 3.

**Table 3:** Details of important simulation parameters used in this work.

Parameter	Value
Data rate (each DL and UL channel)	20 Gb/s
Optical power (each QPSK TX)	25 dBm
Frequency of sinusoidal RF signal	10 GHz
Extinction ratio of DD-MZM	30 dB
Range (each UWOC channel)	25 m
Gain (each optical amplifier)	30 dB
NF (each optical amplifier)	4 dB
Absorption coefficient (each UWOC channel)	0.0405 m^−1^
Scattering coefficient (each UWOC channel)	0.0025 m^−1^
Coupling efficiency	90%
Bandwidth (each WDM drop component)	50 GHz
Bandwidth DEMUX	50 GHz
Responsivity of PIN photodetectors	0.9 A/W
Power density of thermal noise	100 × 10^−24^ W/Hz
Cut-off frequency of ELPFs	0.75 GHz

## 5 Ring failure model at UWOC level

To quantitatively assess the robustness of the proposed UWOC ring architecture, we define a failure model at the UWOC link level. The model considers a single UWOC link between the CU and OADN-1 experiences complete signal loss due to any possible reason such as malfunctioning of telescopes, misalignment due to water currents, or temporary obscuration by marine life. The proposed architecture incorporates a redundant UWOC link configuration between the CU and OADN-1 to enhance reliability as shown in Fig 3. This architecture employs two independent and spatially separated optical paths, a primary UWOC link and a secondary UWOC link each with dedicated transmitter and receiver telescopes. The combined optical signal coming from pulse carver terminates at a Y-select optical switch at OADN-1, which dynamically routes traffic based on link availability. A binary control signal (CS) governs the switching operation. When CS = 1, the switch selects the primary UWOC link for normal data transmission. When CS = 0, indicating primary link failure, the switch immediately shifts the data transmission to the secondary UWOC link. This automatic protection switching ensures uninterrupted connectivity despite individual link failures, providing a concrete implementation of the failure recovery mechanism analyzed in this work.

In long-term underwater deployments, optical components are subject to environmental adverse effects such as pressure, corrosion, bio-fouling, and mechanical misalignment. When properly encapsulated and pressure-rated underwater optoelectronic components are used, the system can achieve mean-time-to-failure (MTTF) values of several years. The proposed ring topology enhances overall system reliability by providing an alternate transmission path, thereby reducing sensitivity to isolated component or node failures. The OADNs in the proposed architecture do not require fast or dynamic active switching. Instead, fixed add-drop functionality and always-on bidirectional transmission are employed, limiting the number of active elements deployed underwater. This approach aligns with the design philosophy of passive or semi-passive ring-based optical access networks such as NG-PON2.

The proposed UWOC architecture supports ring-based protection by exploiting bidirectional signal propagation. Conceptually, a 1 + 1 protection scheme is assumed, where both clockwise and counter-clockwise paths are permanently available, and traffic is recovered from the preferred direction under normal operation. In the event of a node or UWOC span failure, traffic can be logically redirected to the counter-propagating path without requiring dynamic switching at the underwater OADNs. Failure detection may be achieved at the central unit through loss-of-signal, received power monitoring, or BER thresholding. Since protection paths are pre-provisioned, switching times are expected to be on the order of milliseconds to seconds, which is adequate for reliability-driven IoUT scenarios. The protection mechanism does not alter the physical-layer transmission characteristics analyzed in this work. Detailed control-plane protocols and optimization of protection strategies are beyond the scope of the present study and are left for future investigation.

## 6 Results and discussion

Following points are assumed to analyze the performance of DL and UL channels in proposed UWOC ring.

- All OADNs are assembled as anchored nodes above the sea bed and distributed in a ring separated by a fixed distance of 25 m.
- LOS connectivity is assumed throughout the ring among all communicating units, such as CU and OADNs.
- Geometrical loss is considered in DL and UL UWOC channels.
- Thermal noise of photodetectors is ignored due to low water temperature.
- Size and density of underwater bubbles generated are assumed same.
- FEC target BER of 3.8 × 10^−3^ is considered.
- Average BER and Q factors values based on quadrature (Q) and In-phase (I) components are used.
- DL channel-1, 3, and 5 are randomly chosen to discuss the BER performance while channel-2 and 4 are selected to further elaborate the performance with eye-diagrams.
- UL channel-2 and 4 are randomly chosen to discuss the BER performance while channel-1, 3, and 5 are selected to further elaborate the performance with eye-diagrams.

To evaluate the performance of DL and UL channels in the proposed ring topology based UWOC architecture, we have considered different underwater bubble generation rates in OptiSystem. In practical scenario, an adjustable air flow rate pump is used to generate the required underwater bubble rate. BER results are obtained for both DL and UL channels which are measured in OptiSystem using the statistical parameters of the eye-diagrams of the received optical signals. The minimum value of optical power at a photodetector required to achieve a target BER is called receiver sensitivity.

**Fig 7:**
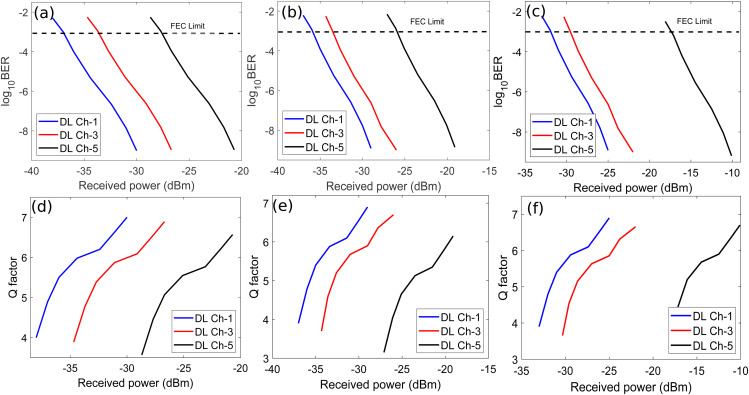
BER versus received optical power plots of DL channel-1, 3, and 5 for (a) Weak bubbling (b) Medium bubbling (c) Strong bubbling and Q factor plots of DL channel-1, 3, and 5 for (a) Weak bubbling (b) Medium bubbling (c) Strong bubbling.

Fig 7 shows the BER and Q factor plots of DL channel-1, 3, and 5 against received optical power for weak, medium, and strong bubble generation rates. It may be noticed from Fig 7a that receiver sensitivities of DL channel-1, 3, and 5 are around −36 dBm, −32.5 dBm, and −25.5 dBm, respectively at the FEC limit for weak bubbling. Similarly, Fig 7b shows that receiver sensitivities of DL channel-1, 3, and 5 are around −34.9 dBm, −32 dBm, and −24 dBm, respectively at the FEC limit for moderate bubbling. Moreover, Fig 7c depicts that receiver sensitivities around −30 dBm, −28 dBm, and −16 dBm are observed for DL channel-1, 3, and 5, respectively for strong bubbling. The system experiences progressive degradation with increasing bubble generation rates. The reason of observed trend in receiver sensitivities of DL channels for all three bubble generation rates can be understood considering the Eq. 7. It may be observed from Eq. 7 that SI depends upon the range of the UWOC channel and turbulence due to underwater bubbles. Therefore, a channel transmitted over longer UWOC link has poor performance compared to the channel propagating over a shorter UWOC link. Fig 3 shows that the DL channel-1 covers the shortest transmission distance of 25 m, channel-3 covers the distance of 75 m, and channel-5 covers the longest distance of 125 m. Therefore, the channel sensitivities are representative of the distance covered over UWOC link. It is also evident from Fig 7a and Fig 7c that overall penalties of 6 dB, 4.5 dB, and 9.5 dB have been observed in received optical power for DL channel-1, 3, and 5 considering weak and strong bubbling, respectively. The system experiences the worst performance degradation relative to the FEC limit for channel-5 as shown in Fig 7c. Channel-5 achieves BER limit at approximately −17 dBm, requiring 10 dB additional power compared to weak bubbling conditions. Fig 7d, 7e, and 7f clearly illustrate that the Q factor values for DL channel-1, 3, and 5 improve with higher received optical power at photodetectors. This trend may be attributed to a fundamental trade-off that achieving a high Q factor, requires increased optical power at the photodetector which consequently limits the UWOC range due to high underwater attenuation.

**Fig 8:**
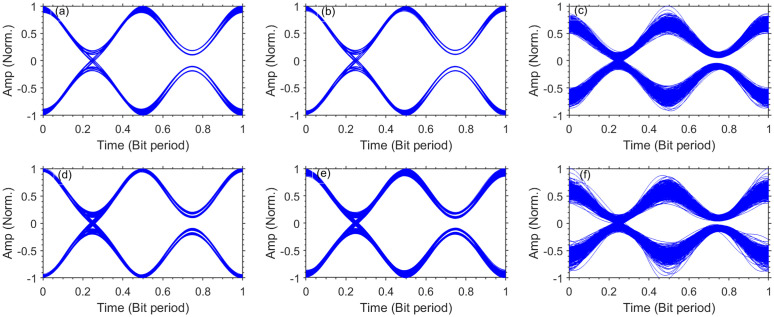
Normalized eye-diagrams for different bubble generation rates. **(a)** DL channel-2, weak bubbling **(b)** DL channel-2, moderate bubbling **(c)** DL channel-2, strong bubbling **(d)** DL channel-4, weak bubbling **(e)** DL channel-4, moderate bubbling **(f)** DL channel-4, strong bubbling.

**Fig 9:**
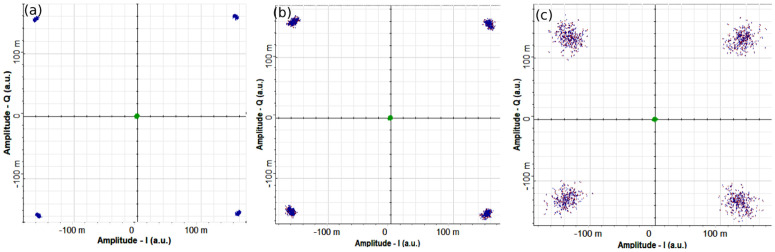
Constellation plots of DL channel-4 for. **(a)** Weak bubbling **(b)** Moderate bubbling **(c)** Strong bubbling.

To further explain the performance of DL channels, we have obtained the eye-diagrams of channel-2 and 4 for different underwater bubble generation rates as shown in Fig 8. It is evident that eye-diagrams of both channels degrade and narrow due to intensity variations on increasing the bubble generation rate. Furthermore, due to propagation over a longer UWOC channel, the eye-diagrams for channel-4 are more degraded and closed than channel-2 for all bubble generation rates. However, the eye-diagrams exhibit enough opening which result into easier discrimination between ’0’s and ’1’s at the receiver. Fig 9 shows the electrical constellation plots of DL channel-4 obtained for different bubble generation rates. It is clear that the constellation points get radiated with increasing the bubble generation rates.

However, the constellation points are well-separated, facilitating straightforward symbol decision at the receiver.

**Fig 10:**
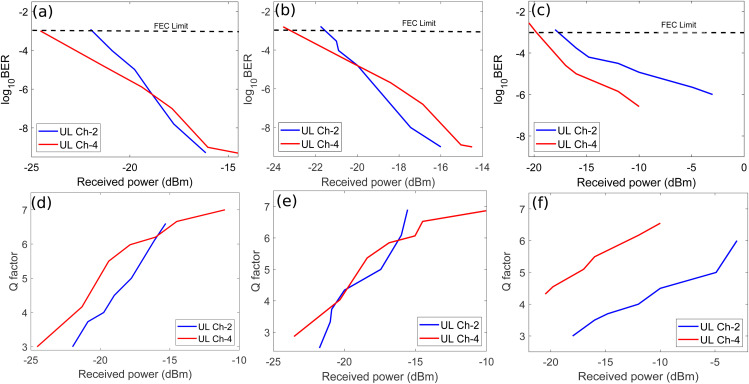
BER versus received optical power plots of UL channel-2 and 4 for (a) Weak bubbling (b) Medium bubbling (c) Strong bubbling and Q factor plots of UL channel-2 and 4 for (a) Weak bubbling (b) Medium bubbling (c) Strong bubbling.

Fig 10 shows the BER and Q factor plots of UL channel-2 and 4 against received optical power for weak, medium, and strong bubble generation rates.

It may be noticed from Fig 10a that receiver sensitivities of UL channel-2 and 4 are around −22 dBm and −24.5 dBm, respectively at the FEC limit for weak bubbling. Similarly, Fig 10b shows that receiver sensitivities of UL channel-2 and 4 are around −21 dBm and −23 dBm, respectively at the FEC limit for moderate bubbling. Moreover, Fig 10c depicts that receiver sensitivities around −17 dBm and −20 dBm are observed for UL channel-2 and 4, respectively considering strong bubbling. The reason of observed trend in receiver sensitivities of UL channels for all three bubble generation rates can be understood again by referring to Eq. 7. We have already discussed above that SI depends upon the range of the UWOC channel and turbulence due to underwater bubbles. Therefore, a channel transmitted over longer UWOC link has poor performance compared to the channel propagating over a shorter UWOC link. Fig 3 shows that the UL channel-2 covers the longer transmission distance equals to 100 m while channel-4 covers the shorter distance of 50 m. It is also evident from Fig 10a and Fig 10c that overall penalties of 5 dB and 4.5 dB have been observed in received optical power for UL channel-2 and 4 considering weak and strong bubbling, respectively. The system experiences the worst performance degradation relative to the FEC limit for channel-2 as shown in Fig 10c. Channel-2 achieves BER limit at approximately −17 dBm, requiring 5 dB additional power compared to weak bubbling conditions. Fig 10d, Fig 10e, and Fig 10f clearly illustrate that the Q factor values for UL channel-2 and 4 improve with higher received optical power at photodetectors. This trend may again be attributed to a fundamental trade-off that achieving high Q factor, requires increased optical power at the photodetector which consequently limits UWOC range due to high underwater attenuation as we have already discussed earlier.

**Fig 11:**
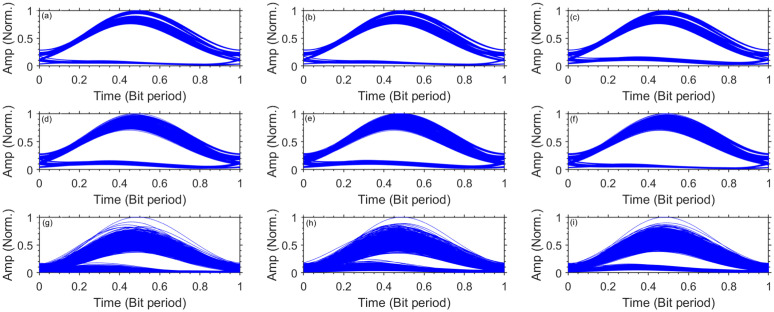
Normalized eye-diagrams for different bubble generation rates. **(a)** UL channel-1, weak bubbling **(b)** UL channel-3, weak bubbling **(c)** UL channel-5, weak bubbling **(d)** UL channel-1, moderate bubbling **(e)** UL channel-3, moderate bubbling **(f)** UL channel-5, moderate bubbling **(g)** UL channel-1, strong bubbling **(h)** UL channel-3, strong bubbling **(i)** UL channel-5, strong bubbling.

To further explain the performance of UL channels, we have obtained the eye-diagrams of channel-1, 3, and 5 for different underwater bubble generation rates as shown in Fig 11.

It is evident that eye-diagrams of all three channels degrade and become narrow due to intensity variations on increasing the bubble generation rate. Furthermore, due to propagation over a longer UWOC channel, the eye-diagrams for channel-1 are more degraded and closed than channel-5 for all bubble generation rates. However, the eye-diagrams exhibit enough openings which result into easier discrimination between ’0’s and ’1’s at the receiver. While the system achieves the FEC target BER for all turbulence regimes, the transmission of DL and UL channels comes with significant power penalties, particularly under strong bubbling and over longer transmission distances. The observed 6–10 dB penalties for DL channels and 4.5–5 dB penalties for UL channels indicate that operation under intense turbulence substantially erodes the link margin and power efficiency. Therefore, practical deployment in such conditions would require either adaptive power control, reduced link lengths, or advanced turbulence compensation to ensure reliable and efficient performance.

**Fig 12:**
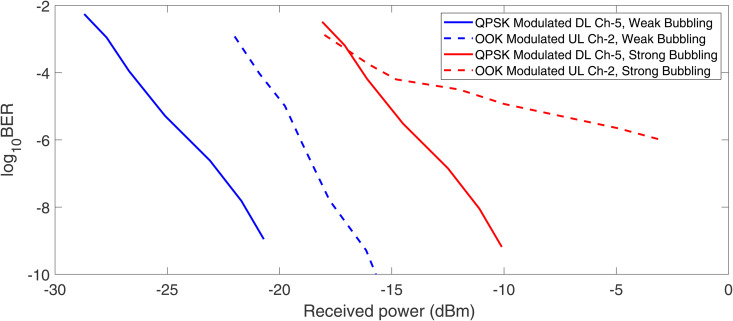
BER performance comparison of QPSK and OOK modulated DL and UL channels, respectively in weak and strong bubble generation rates.

In Fig 12, the performance of QPSK modulated DL channel-5 has been compared with OOK modulated UL channel-2 for weak and strong bubble generation rates. It can be observed that for any given level of received optical power, QPSK modulated channels achieve a significantly lower BER values compared to OOK modulated channels, highlighting the better performance. Moreover, the performance of QPSK channel degrades significantly under strong bubble conditions, as indicated by the shifting of the curve to the right which indicates a higher power requirement to maintain the same BER value. However, this detrimental effect is more significant for OOK modulated channels, underscoring QPSK’s advantage in strong bubbling conditions. For performance benchmarking with state of the art published works, we have compared the main results of proposed architecture with related past published works as discussed in Table 1.

It is clearly evident from Table 4 that the proposed work outperforms the related past works on the basis of channel data rate, total range, and spectral efficiency.

**Table 4:** Performance benchmarking with state of the art published works.

Study	Rate	Modulation	Range	Directionality	Wavelength
[9]	1.2 Gb/s	OOK	30 m	Full-duplex	525 nm
[10]	10 Mb/s	OOK	105 m	Full-duplex	450 nm
[12]	25 Mb/s	4QAM	50 m	Duplex	450 nm
[13]	0.5 Mb/s	OOK	10 m	Full-duplex	470 nm
Proposed	20 Gb/s	QPSK/OOK	150 m	Full-duplex	532 nm

**Fig 13:**
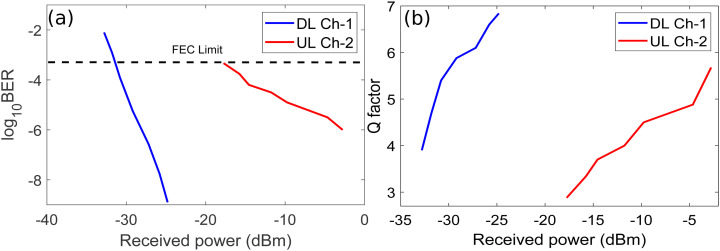
(a) BER versus received optical power plots of DL channel-1 and UL channel-2 for strong bubbling considering secondary UWOC link (b) Q factor plots of DL channel-1 and UL channel-2 for strong bubbling considering secondary UWOC link.

To analyze the performance of DL and UL channels considering the predefined failure model with the CS = 0 (selecting the secondary UWOC link), Fig. 13 shows the BER and Q factor plots of DL channel-1 and UL channel-2 for strong bubbling. For simplicity and as a proof of concept, we randomly select only one DL channel and one UL channel under the strong bubbling condition while keeping all other parameters unchanged.

For both DL Channel-1 and UL Channel-2 under strong bubbling conditions, the BER and Q factor plots exhibit negligible performance deviation compared to DL Channel-1 and UL Channel-2 under strong bubbling conditions with the CS = 1 (selecting the primary UWOC link). This indicates that the secondary link, with independent transmitter and receiver telescopes provides same signal integrity and turbulence resilience. The observed parity in performance metrics confirms that the automatic protection switching mechanism maintains seamless service continuity without introducing additional signal degradation, thereby validating the redundancy scheme’s capability to uphold network reliability during primary link failures. To elaborate the connectivity and path inflation after failure, the ring architecture employs OADNs, where each OADN drops one DL channel and adds one uplink UL channel. In the event of a single OADN failure, the remaining four OADNs maintain connectivity to the CU, resulting in 80% node survivability while preserving full optical layer functionality for the operational nodes. When the secondary UWOC link protection mechanism is activated, the architecture guarantees seamless connectivity for all OADNs to the CU, achieving 100% node survivability even under primary link failure conditions. The secondary link ensures that data transmission is re-routed without changing the hop count or the accumulated link range, thereby maintaining consistent latency and signal quality.

For more information about raw data required to build the various plots, see S1 Data.

## 7 Conclusions

We implemented a spectral efficient and high bit rate underwater wireless optical communication ring transmitting full-duplex data to five different optical add-drop nodes without using optical carriers for uplink transmission. The combined optical signal generated at the central unit composed of five quadrature phase shift keying downlink channels, each at the rate of 20 Gb/s was transmitted towards the nodes. Downlink baseband data recovered at respective nodes and same optical carrier was re-used for on-off keying modulated uplink data transmission, each at the rate of 20 Gb/s towards central unit. To implement the full-duplex transmission in the ring, a pulse carver based on dual drive Mach-Zehnder modulator was employed. Underwater bubbling induced turbulence was modelled by Gamma-Gamma distribution. The performance of downlink and uplink transmissions were evaluated using Bit-error rate plots for different underwater bubble generation rates. The results indicated that target forward-error correction Bit-error rate limit of 3.8 × 10^−3^ was achieved for all downlink and uplink channels for different underwater bubble generation rates. Failure modeling demonstrated 80% and 100% node connectivity with CU for single OADN and primary UWOC link failure through automatic link redundancy, respectively while maintaining the path inflation.

## Supporting information

S1 Data Raw data required to build the BER and Q factor plots. (XLSX)
